# Recruitment Strategies and Colony Size in Ants

**DOI:** 10.1371/journal.pone.0011664

**Published:** 2010-08-04

**Authors:** Robert Planqué, Jan Bouwe van den Berg, Nigel R. Franks

**Affiliations:** 1 Department of Mathematics, VU University Amsterdam, Amsterdam, The Netherlands; 2 School of Biological Sciences, Bristol University, Bristol, United Kingdom; University of Utah, United States of America

## Abstract

Ants use a great variety of recruitment methods to forage for food or find new nests, including tandem running, group recruitment and scent trails. It has been known for some time that there is a loose correlation across many taxa between species-specific mature colony size and recruitment method. Very small colonies tend to use solitary foraging; small to medium sized colonies use tandem running or group recruitment whereas larger colonies use pheromone recruitment trails. Until now, explanations for this correlation have focused on the ants' ecology, such as food resource distribution. However, many species have colonies with a single queen and workforces that grow over several orders of magnitude, and little is known about how a colony's organization, including recruitment methods, may change during its growth. After all, recruitment involves interactions between ants, and hence the size of the colony itself may influence which recruitment method is used—even if the ants' behavioural repertoire remains unchanged. Here we show using mathematical models that the observed correlation can also be explained by recognizing that failure rates in recruitment depend differently on colony size in various recruitment strategies. Our models focus on the build up of recruiter numbers inside colonies and are not based on optimality arguments, such as maximizing food yield. We predict that ant colonies of a certain size should use only one recruitment method (and always the same one) rather than a mix of two or more. These results highlight the importance of the organization of recruitment and how it is affected by colony size. Hence these results should also expand our understanding of ant ecology.

## Introduction

Many organisms rely on strength in numbers [1]. Seabirds often nest synchronously and in huge colonies to reduce the chances that their offspring will be killed by predators [2]; wildebeest mass together, to reap similar selfish benefits, during migration across the African plains [3]. Animals cooperating with one another to increase their collective chances of survival may also need to regulate how to share information such as the location of food. The reliability of such protocols often depends on the number of individuals involved [4]. Colonies of bacteria consist of such large numbers that they can rely on anonymous chemical signals to aggregate and form spawning bodies to reproduce [5]. Behavioural displays such as those used by wolves, dolphins or monkeys are only effective when they are performed in front of conspecifics, and hence in relatively small groups (see [6], [7], and many references therein). Such examples highlight links between internal structures, such as division of labour and communication protocols, and observed group sizes. One taxon displaying tremendous variation both in colony sizes (over six orders of magnitude) and internal organization are the ants.

Ants have achieved great ecological success both in terms of their diversity and their biomass [8], with over 12,500 described extant species (http://antbase.org). Ant diversity is associated with the variety of resources they consume, and their broad range of nesting habits, patterns of colony organization and life histories. Ants often have vastly greater biomasses than solitary insects that mostly consume the same resources [8]–[10]. One likely explanation for their abundance is that ants gain efficiencies both through a division of labour and the ability to summon nestmates to resources that they can therefore dominate and exploit fully [11]. This highlights the importance of their recruitment systems. Wilson [12] has neatly defined ant recruitment as “communication that brings nestmates to some point in space where work is required.”

Clearly recruitment and resource distributions should be linked and accordingly Hölldobler and Wilson [13] point out that “the recruitment strategy [of ants] makes little sense except with reference to the ecology of the species.” Nevertheless, these authors also suggested that the sophistication of the chemical recruitment that ants exhibit correlates positively with the size of colonies. Indeed, Beckers *et al.* [14] have shown that there is a correlation between mature colony size in ants and the recruitment methods they use during foraging. Recruitment, of course, involves interaction between ant workers, and the various methods differ in the way these interactions take place. Hence, at least some of the correlation between recruitment method and colony size may be explained by considering which methods can be used effectively at a certain colony size. In this paper we focus on direct links between colony size and recruitment strategy by considering how build up of recruiter numbers using different strategies depends on the size of the colony.

Certain ants do not use recruitment during foraging and have so-called solitary foragers. Others perform tandem runs in which one ant leads a single nestmate to a target. In group recruitment, one ant worker may lead a group of a few to perhaps thirty ants to a goal. Still other ants use recruitment pheromone trails that channel often copious flows of ant traffic from the nest to important resources. These methods loosely correlate with mature colony sizes such that solitary foraging is typically associated with smaller workforces, tandem running occurs in small to medium sized colonies, group recruitment is deployed by colonies of medium size and recruitment pheromone trails are associated with large colonies [14].

Many recruitment methods used by ants involve positive feedback as recruited ants go on to recruit yet more nest mates: such is the case for methods as different as tandem running [15] and pheromone scent trails [16]. Such positive feedback also raises issues related to reliability and colony size.

Here we explore possible relationships between recruitment methods and colony size in ants, highlighting some largely unexplored issues. Some ant species are capable of performing more than one recruitment method, such as *Camponotus socius* [17], two *Formica* species [18], two *Tetramorium* species [19], [20] and two *Aphaenogaster* species [21]. If we consider ants that are capable of more than one recruitment method, can we predict which recruitment method is likely to be used? Does the recruitment method that ants use result from a relationship between colony size and reliability of recruitment, defined as the per capita probability for a recruitment act to fail? (Note that we are not considering foraging efficiency here, but merely ‘recruitment competitiveness’, i.e., how one recruitment strategy compares to another when both are competing for recruits.) Are different recruitment methods likely to be mutually exclusive at any given colony size? We explore these issues with suitable mathematical models.

## Methods

### Modelling

Our models describe the build up of recruiter numbers using two strategies, group recruitment and pheromone trails (we will consider tandem running as a special case of group recruitment, with group size 1; i.e., one ant follows the leader). This type of model may also apply to colonies performing no recruitment behaviour, or performing just one recruitment behaviour. As we elaborate in the Discussion, some species lineages seem to have evolved pheromone trails first and later acquired tandem running, whereas in other taxa this is the other way around [22]. Hence, if one of these is to be deployed as a new strategy, their first use might be in a context of the other recruitment strategy. The “competition between recruitment methods” we model may thus be a rare phenomenon, but could be important on an evolutionary scale, and may be applied to understand differences between species.

Consider a colony of 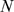 ants, each of which is assumed to be capable of performing either recruitment method. Each ant decides randomly which method to use upon being recruited to a target such as food. The ants are assumed not to switch strategy whilst committed to one, and are equally inclined to perform either method. Both recruitment methods are modelled by considering simple positive feedback mechanisms and rates of failure.

For the build up of pheromone trail recruiters, our model is based on the recent study of phase transitions in foraging strategies by Pharaoh's ants *Monomorium pharaonis* by Beekman *et al.* [23]. Denote the number of ants laying pheromone trails by 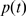, and the number of ants involved in group recruitment as followers by 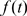 and as leaders by 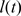. The remaining ants are uncommitted workers, denoted by 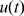. The change per unit time in the number of pheromone laying ants is assumed to be given by
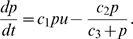
(1)


Here, 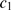 is the per capita rate at which individual ants using pheromone trails recruit ants not currently engaged in any recruitment, 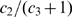 is the maximum per capita rate at which ants fail to keep following the trail (attained when 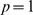, the minimum number of ants laying a trail). This rate function reflects that pheromone trails work poorly when numbers of trail laying ants are low, but work efficiently when these numbers are high. We have assumed that more ants on trails means stronger trails, and therefore stronger recruitment to it, and also that the pheromone trail dynamics are assumed to be fast with respect to the recruiter number dynamics. A more detailed model would involve separate equations for ants and pheromone concentrations, but would make the analysis much more involved.

In group recruitment, leader ants guide follower ants to the recruitment target. If a tandem group reaches its destination succesfully, the following ants become group leaders and start leading their own tandems. If a group recruitment act fails, there are two possible modelling choices. Either we assume that the ants following the leader remain committed to group recruitment for some time and team up with leader ants to try again, or we assume that those ants simply become uncommitted ants again. This latter assumption follows more closely the assumption with regard to pheromone trails, where ants losing a trail become uncommitted again. This choice also makes the ensuing model simpler as we have one class of ants less, the ‘follower ants not currently following a group leader’. For these two reasons, we make the second assumption. We do not expect the solutions to behave qualitatively different if the other choice were made.

To make new groups of recruits at some moment in time, there must be ants currently classified as leaders but not actively leading a group. This number of uncommitted or free leaders 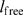, is equal to 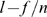. Free leader ants form groups of recruits at the nest, which are formed from uncommited ants 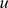, at a rate 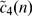. The change in numbers of follower ants is then proportional to 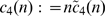. We assume that new follower ants are recruited at a rate proportional to both numbers of free leader ants and uncommitted ants.

Assuming, finally, that the number of followers decreases at a fixed rate at which followers cease to follow a leader, these assumptions lead to
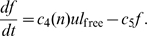
(2)


The rate 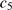 is in fact the sum of two processes: either follower ants reach their destination successfully through group recruitment and then become leaders, which we assume happens with probability 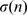, or they do not. Free leaders are also assumed to stop recruiting for a food source at a fixed rate, 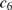, say, so that
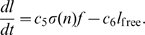
(3)


To complete the model, we also state how the number of uncommitted ants change as the result of the two recruitment processes,
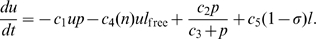
(4)


Equations (1)–(4) constitute the main model. Since 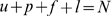, it suffices to consider the dynamics only of 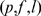, i.e. equations (1)–(3). As initial conditions we take a colony which is about to start recruiting to a food source, so throughout we set 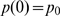, 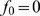, 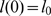, with 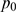 and 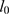 both positive but small.

Within this model framework, tandem running is seen as a special case of group recruitment with group size 1. We are aware, as explained in detail in the Discussion, that there are important functional differences between tandem running and group recruitment. However, all that matters in this model is that the per capita failure rate 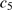 is independent of the number of groups being recruited (as opposed to scent trails).

The main analysis we will perform on this model is to understand which steady state (if any) will be reached when the colony starts with a few recruiters of either type, and in particular, how the stability of such steady states change with colony size 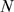. We are thus not interested here in any optimality arguments (which recruitment strategy would give the highest yield, for example), nor do we model any real harvesting of food. These models focus entirely on the build up of recruiter numbers in behaviourally flexible colonies.

## Results

First we may assume that 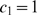 by rescaling time and changing all other constants appropriately. The model has five equilibria, 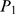 through 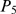, which all lie in a plane
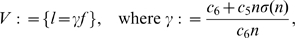
and solutions always approach one of these in the long run. Since at steady state 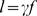, we express these steady states as pairs 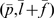. The following constants will play an important role in the description of the steady states, and their stability:
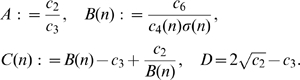
The equilibria are given as pairs 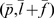.

**Figure 1:**
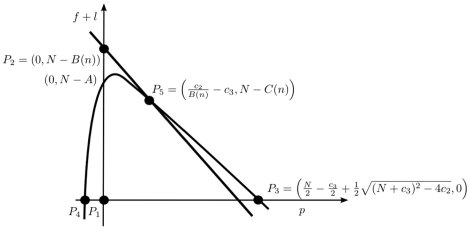
Overview of the position of steady states. The five equilibria are sketched in the 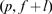 plane to indicate their relative positions for a particular choice of parameter values. Coordinates of the most important steady states have been indicated. The diagonal straight line through 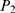 is an isocline for the 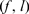 dynamics, and the curved line through 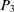, 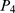 and 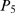 is an isocline for the 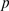 dynamics. This latter line intersects the 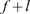 axis at 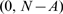.

- One stationary point for ‘solitary foraging’ (i.e., no recruitment at all), 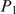, which is the origin;
- one for group recruitment only,
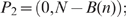

- two for pheromone trails only, which exist when 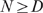:
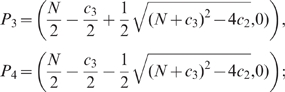

- one in which ants use both group recruitment and pheromone trails,
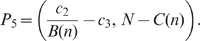

Depending on parameter settings, 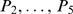 may be biologically unrealistic (and 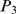 and 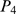 exist only when 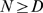), so solutions cannot always approach all five equilibria when starting out with small positive initial conditions. See Figure 1 for an overview of the positions of the different equilibria.

### Stability of equilibria

Apart from the three trivial isoclines (the 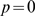 plane, the 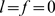 line, and the 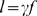 plane), there are two nontrivial isoclines: one (a group recruitment isocline) indicates where the number of ants involved in group recruitment and the number of uncommitted ants are in balance with respect to each other, while the other (a pheromone recruitment isocline) indicates where the number of ants involved in pheromone recruitment and the number of uncommitted ants are in balance with respect to each other. The points where a group and a pheromone isocline intersect are equilibria. At each steady state, we calculate the Jacobian matrix and determine its eigenvalues to understand its linear stability. We are mainly interested how these stability properties change when we vary colony size 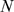, and which steady state attracts solutions starting near the origin.

As shown in Figure 1, the non-trivial pheromone isocline intersects the 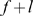 axis at 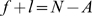 whereas the non-trivial group recruitment isocline intersects this plane at the 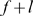 axis at 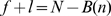. The constants 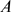 and 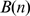 thus determine the ordering of these two intersection points. This ordering, as we will see, determines much of the stability of the equilibria.

There is another ordering of points, where the two non-trivial isoclines intersect with the 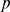-axis. The 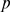-isocline intersects the 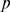-axis twice when 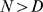, at the 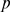-coordinates of 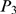 and 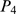. The other isocline intersects the 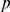-axis at 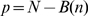. The constant 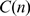 is a measure for the distance between the intersections of the two isoclines with the 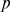-axis, 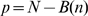 and the 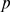-coordinate of 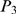: 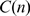 switches sign precisely when this distance vanishes. The ordering between 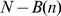 and the 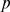-coordinate of 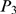 shall also influence the stability of the steady states.

We now describe the stability properties of these five equilbria, using 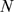 as our main parameter.

First we consider the trivial steady state, 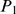. It has three eigenvalues; one is equal to 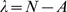, and two that change sign at 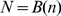, both becoming positive for 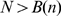. The ordering between 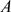 and 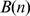 thus directly determines which eigenvalue becomes positive first, as 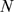 increases.

For 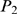, we have one eigenvalue 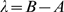, and again a pair of eigenvalues changing sign at 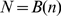, but now they become negative for 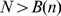. So if 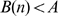, then 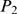 takes over stability from 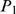 as 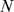 passes 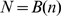.

The third (and fourth) steady state only exists if 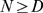, and we need to consider two cases. Note that 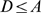 for all 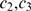.

If 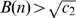, then the top of the pheromone isocline lies above the group recruitment isocline. Now 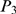 is locally stable for all 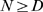. 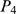 is unstable for small 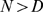, passes through the origin at 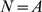. It remains unstable througout. 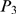 is reachable for solutions starting near the origin only when 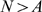. Note that in this case, 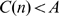.

If 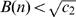, then for 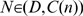, 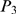 lies to the left of 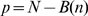, and is unstable. Two eigenvalues of 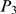 change sign at 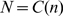, making 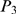 locally stable. If 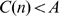, then 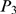 is not reachable from the origin for 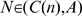, but as 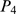 passes the origin at 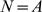, 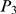 is a stable attracting steady state for solutions starting at the origin. If 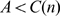, 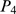 has passed the origin before 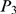 becomes stable, so 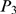 is stable and attracting for all 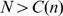.

In summary, 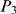 is locally stable and attracting for solutions starting near the origin if and only if 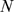 is larger than both 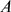 and 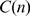.

Note that from these results it follows that minimum colony sizes are needed for 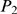 and 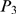 to be biologically realistic. The value of 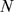 where this happens (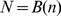 and 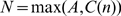 respectively) coincides with these steady states becoming stable and attracting.

Finally, the mixed steady state 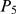 lies in the first octant if 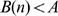 and 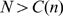. The lowest order constant of the characteristic equation is the determinant of the Jacobian at this point, and since the highest order term is 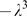, a positive determinant means a positive root must exist. This determinant is positive if and only if 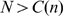. This coincides with an increase of stability of 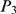. In all, whenever 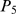 lies in the first octant, it is unstable.

### Three cases

**Figure 2:**
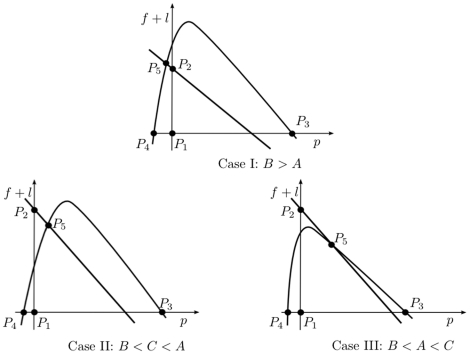
Isoclines and equilibria for the three cases. The three cases are explained in the text. Importantly, changing 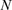 merely means that the position of the 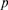-axis changes relative to the other isoclines: increasing 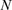 means lowering the 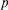-axis. The difference between Case 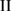 and 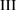 is that the steady state 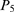 lies above (Case 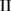) or below (Case 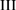) the intersection point of this 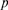 isocline with the 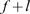 axis. This results in a different bifurcation sequence. Detailed explanations of these bifurcations are given in the text.

**Figure 3:**
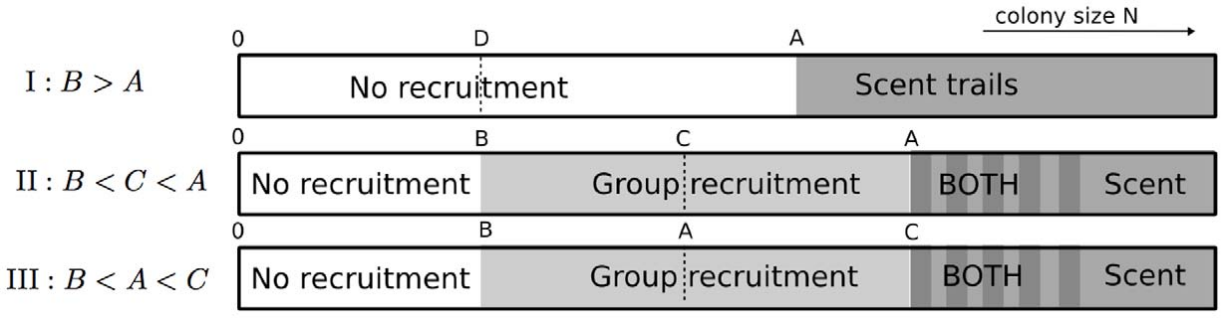
Comparing the three bifurcation scenarios. As colony size 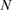 increases, three successions of recruitment methods may take place, indicated by 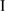, 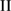 and 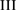. Depending on the relative magnitude of parameters 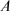, 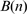 and 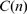, the ordering of the different methods changes. Note that in Case 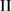 and 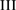, there are two attracting steady states for 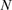 large, but as 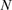 increases pheromone (scent) trails will most likely be used. See the text for details.

We can summarise these results into three possible bifurcation scenarios for solutions starting near the origin. See Figure 2 for an illustration of the different isocline arrangements, and Figure 3 for a sketch of the bifurcation sequences in these three cases.

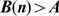
. Then 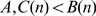. For 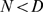, 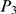 does not exist, and 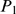 is the only attractor. If 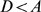, then for 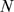 between 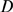 and 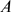, 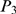 does exist and is stable, but cannot be reached from the origin. For 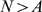, 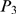 is the unique attractor.

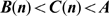
. This is equivalent to 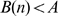 and 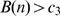. For 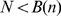 the origin is stable but loses stability at 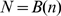 to the group recruitment steady state 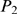. Now, as 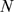 passes first 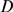, 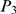 (and potentially 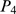) become biologically relevant. 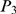 is potentially stable, depending on the sign of 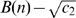, but not attracting. As 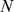 passes 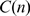, the mixed steady state 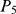 enters the first octant but is unstable, conferring stability to 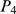 (but remains unstable). At 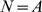, 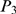 is now also reachable from points close to the origin. So for 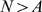, both 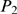 and 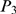 are stable and attracting steady states from the origin, and the parameter settings decide which one is attracting for practically all orbits starting near the origin. The mixed steady state 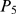 is unstable whenever it is biologically realistic.

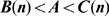
. This is equivalent to 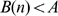 and 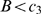. For 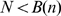 the trivial steady state 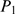 is the only stable equilibrium. As 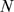 passes 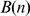, 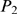 enters the first quadrant and takes over stability from 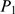. As 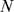 passes first 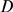 and then 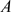, 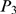 enters but remains unstable until 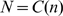 when the mixed steady state 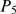 becomes biologically realistic. Now both 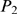 and 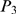 are stable and reachable for 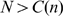, and 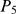 is unstable. Again, which of the two stable steady states 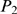 and 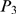 is attracting for most orbits starting near the origin depends on particular parameter settings.

Biologically, these cases may be interpreted as follows. In Case I, small colonies use only solitary foraging, whilst larger colonies use pheromone trails. In Cases II and III, small colonies forage solitarily, colonies of intermediate size use group recruitment, and large colonies use either group recruitment or pheromone trails. The precise sequence of events as colony size increases differs between case II and III, but the observed pattern is the same.

When both 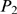 and 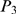 are stable, only one will be the dominant strategy, in the sense that nearly all orbits starting in a neighbourhood of the origin end up in this dominant steady state. By considering the dependence on 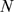 of the eigenvalues at the origin, it can be shown that the dominant eigenvector switches with increasing 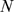: for small 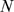 group recruitment is the dominant strategy, while pheromone trails dominate for large 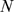. This is in agreement with the loose correlation between colony size and recruitment strategy found by Beckers *et al.* [14].

Having explicitly modelled group recruitment with group size 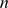, rather than mere tandem running, say, it would now of course be interesting to understand how 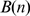 (and hence 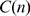, which is a function of 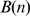) changes as a function of 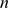. At this moment, however, we cannot infer much about the possible shape of these two parameter combinations. Recall the definitions of 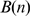 and 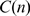. Although it is plausible that the rate at which groups of size 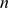 are assembled, 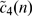, should be decreasing in 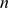, this does not allow us to infer that 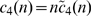 decreases as well. Whether 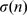 is decreasing or increasing is also unknown at present. Nevertheless, these parameters should be open to experimentation. This would provide interesting additional insight into why species with intermediate colony sizes use group recruitment more often than smaller or indeed larger ones.

In the absence of information on the behaviour of 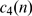 and 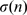, we can at least show one additional result under certain assumptions. Let us assume that the rate at which groups are assembled decreases sufficiently in 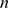 that 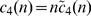 is still decreasing in 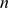. Then recruiting in larger groups will only work if the per capita probability of reaching the food source 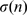 increases with 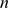. If we make slightly stronger assumptions, namely that 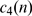 is also convex, with limit 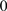, and that 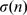 is concave with limit 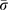, then with any choice of positive rate constants and group size 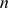, there exists a minimal colony size for this recruitment system to function. If 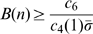 for all 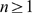, then group recruitment cannot work at any colony size.

As briefly mentioned in the Methods section, this model also applies to ant species which only use one strategy. All we need to do to analyse this situation is to restrict our class of initial conditions. Rather than starting with a few ants for each strategy, we could start with only a few ants using just one strategy, thus ensuring that the dynamics remains confined to the 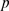-axis or the 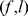 plane. We can still deduce that minimal colony sizes are needed for each strategy, and these are indeed the same as for the complete model. The full model adds to this by considering how competition for recruits using two methods results in a final ‘chosen’ strategy. and indicates that one method is expected to be used after initial recruitment build up.

## Discussion

The main model predictions are the following.

- Within colonies using multiple recruitment methods, only one method is likely to be used consistently. Use of both strategies is never a stable situation.
- For all recruitment methods a certain minimal colony size is required.
- As colony size increases (and all other rate parameters remain fixed), ants should change from solitary foraging to pheromone recruitment, possibly with tandem running and/or group recruitment as an intermediate stage. The parameter combinations that determine the precise sequence are 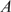, 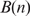 and 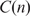. Most importantly, if 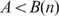, then colonies are expected to switch from solitary foraging to scent trails as colony size increases, but when 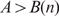, an intermediate stage of group recruitment is predicted. Parameters 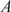 and 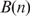 may be interpreted biologically as follows. 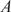 is approximated by 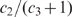, the maximal per capita probability of losing a trail, and 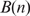 is the number of ants not involved in recruitment at the group recruitment steady state. Hence, if pheromones are very effective and reliable to follow, 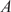 decreases and scent trails are used more readily by ants; if group recruitment actively involves a larger part of the colony, 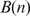 decreases, and group recruitment is favoured more quickly.
- Very large colonies are always expected to use scent trails.

Our modelling and analyses thus suggest that one of the reasons for the observed correlation between different recruitment methods and colony size in ants is that the reliability of the recruitment methods differs as the number of participating ants changes. These predictions do not use any particular knowledge of the rate parameters in the models.

The use of two recruitment methods in one colony, and the resulting exclusion of the employment of both simultaneously, is in effect a biological switch. In biochemical systems such as gene regulatory networks, it is now known, in detail how the enzymes involved must how react with substrates to implement a switch [24], [25]. In eusocial insects such as ants and bees, biological switches have been studied intensively in recent years using both experimental and theoretical approaches, particularly in the context of nest choice [26]–[32]. Here the central questions have been: how can a colony trade speed for accuracy in their decision, and which behaviours contribute to optimizing such a trade-off; and how do they choose only one of many possible paths to a food source [20], [33]. In this paper we have studied a biological switch where the object of choice is not the nest or the food source, but the *strategy* to recruit to such targets.

This correlation is illustrated by many well-known examples. Species with small mature colony sizes, for instance, seem predominantly to use solitary foraging (this is the case in many ponerines, such as *Amblyopone* [34], [35] and in primitive ants such as *Nothomyrmecia* and *Myrmecia* (Hölldobler & Wilson 1990). Species with small to medium colony sizes seem to use tandem running (Beckers *et al.* 1989) and tandem running seems to have evolved independently several times in ants (it occurs in many ponerines, the formicine *Camponotus socius* and in myrmecines such as *Temnothorax*) (Hölldobler & Wilson 1990). Group recruitment occurs in species with arguably slightly larger mature colony sizes. Finally, the use of recruitment pheromone trails is extremely common in ants with large colony sizes, such as *Eciton* and *Dorylus* army ants [36], [37].

Of course, the traditional argument is that these different recruitment methods are associated with resource distributions and other aspects of ecology. Thus it might rightly be claimed that the desert living *Cataglyphis* uses solitary foraging because pheromone trails would quickly evaporate at high temperatures. Alternatively, they might only forage solitarily because the small arthropods they feed on can be retrieved by solitary workers [38]. We do not doubt that many such factors have influenced the solitary foraging habits of *Cataglyphis*—however, reliability might underpin much of the behaviour of these fascinating ants. Indeed, it is absolutely essential for their own survival that *Cataglyphis* workers can navigate reliably and return swiftly to their nest. Given that pheromone trails may be excluded by desert temperatures and substrates, why have they not evolved tandem running? We suggest that one reason is that it is both slow and occasionally unreliable compared to their high grade solitary foraging (see [39] and references therein).

Tandem running occurs in both supposedly primitive and highly derived ant genera and has evolved independently several times [13], [40], [41]. Indeed, the one thing these tandem running ants have in common is small colony size and both small colony size and tandem running may be derived states in for example *Temnothorax*: the ancestors of some extant tandem running species may have had large colony sizes and pheromone recruitment [22]. Franks & Richardson [15] have shown that tandem running in *Temnothorax albipennis* meets all of the criteria of a formal and well established definition of teaching in animal behaviour [42]. It is both a reliable method to take a nest mate to a food source or new nest, and also allows the tandem follower to teach others. The refinement with which tandem running is used in *Temnothorax* exemplifies the importance of high reliability of recruitment in this species [15], [43].

Group recruitment seems to fit beautifully between tandem running and pheromone recruitment. The leader ant is stimulated by the ant immediately behind it, but often also deposits short-lived scent marks allowing more than one ant to follow (Hölldobler & Wilson 1990). Group recruitment seems to be a very reliable method to get a group of ants quickly to a certain goal, but many tandem running ants do not seem to be able to use it, despite their use of scent marks for other purposes (for an example in *Temnothorax*, see [44]).

The efficacy and reliability of pheromone recruitment trails to large colonies is evident given the copious ant traffic that such trails can maintain. Well-known examples include the European black garden ant (*Lasius niger*) [33], Argentine ants (*Linepithema*) [45], fire ants such as *Solenopsis invicta*, and *Eciton* and *Dorylus* army ants [37]. In one trail laying species, the Pharaoh's ants (*Monomorium pharaonis*), it has indeed been found experimentally that a minimum number of ants is needed for these trails to function [23].

As we discussed in the Introduction, some species do use more than one recruitment method. It thus seems plausible that they might use one method more when their colonies are small and favour an alternative after their colonies have become larger. However, we urge caution over the interpretation of the use of pheromone trails, when observed in a particular species. Chemical trails layed by ants are sometimes purely for orientation rather than for recruitment (see for example [17], [46]). So it will be essential to determine if ants are using more than one active recruitment system rather than, say, just orientation trails and tandem running.

A major gap in our knowledge of ant recruitment systems is whether even common species that begin with small colonies and grow to large ones pass through several recruitment methods. One example might be *Amblyopone*. Ito [35] has suggested that the presence or absence of different recruitment systems within this genus may be associated with species-characteristic colony sizes. Evidence at present is circumstantial, with group recruitment occurring in one species of *Amblyopone* with relatively large colonies (about 100 workers) [35], and others working on species with smaller colony sizes reporting no elaborate communication during foraging [34], [47], [48]. But do some species pass through tandem running of group recruitment before starting to lay trails? To put this in even bolder terms: do any ant colonies pass through a colony-level metamorphosis in their recruitment methods as they grow from small cottage industries to huge city states?

One function of this paper is therefore to encourage entomologists to study ant recruitment over a range of colony sizes, especially in species with large mature colony sizes and that use pheromone recruitment trails. It will be intriguing to see if there are gradual transitions in recruitment methods or metamorphoses in growing colonies. It will even be fascinating if any such transitions are not observed, because this might predict that small colonies may grow extremely slowly (if they are using recruitment systems that are inappropriate for their given size) giving larger conspecific colonies a competitive edge because they can recruit so much more effectively. Either way, we would better understand links between recruitment systems and ecology—an important goal as suggested by Hölldobler and Wilson (1990).
